# Identification and Validation of Biomarkers to Predict Early Diagnosis of Inflammatory Bowel Disease and Its Progression to Colorectal Cancer

**DOI:** 10.1007/s10528-024-10917-z

**Published:** 2024-09-26

**Authors:** Farhat Khan, Naaziyah Abdulla, Thea-Leonie du Plessis, Kay Karlsson, Peter Barrow, Brendan Bebington, Liang Gu, Mandeep Kaur

**Affiliations:** 1 School of Molecular and Cell Biology, University of the Witwatersrand, Private Bag 3, Johannesburg, WITS-2050 South Africa; 2 Wits Donald Gordon Medical Centre, Park Town, Johannesburg, 2193 South Africa

**Keywords:** Inflammatory bowel disease, Colorectal cancer, Diagnostic biomarkers, Transcription factors, In silico and wet-lab validation, Predictive markers

## Abstract

Inflammatory bowel disease (IBD) has become a common global health problem as prevalence continues to rise. It is often associated with increased risk of colorectal cancer (CRC) development. Limitations in current IBD biomarker-based diagnosis hinder the accuracy of early detection of CRC progression. Therefore, in this study, we proposed the use of transcription factor (TF)-based biomarkers that can potentially detect the transition of IBD to CRC. Various bioinformatic analysis and online database validations, and RT-qPCR validations were performed to identify possible diagnostic TFs. *RUNX1* was identified as a promising TF that regulates 106 IBD/CRC-related genes. The incorporation of *RUNX1* in combination with currently known IBD biomarkers, *FEV* + *NFKB1* + *RELA*, achieved a comparable sensitivity and specificity scores of 99% and 87%, respectively, while *RUNX1* in combination with known CRC markers, *CEA* + *TIMP1* + *CA724* + *CA199*, achieved a sensitivity and specificity score of 97% and 99%, respectively. Furthermore, a small pilot RT-qPCR-based analysis confirmed a demarcated shift in expression profiles in *CA724, CEA, RUNX1* and *TIMP1* in IBD patients compared to CRC patients’ tissue samples. Specifically, *CA724* is noticeably elevated in IBD, while the levels of *CEA, RUNX1* with *TIMP1* are probable genes that may be employed in discerning IBD progression to CRC. Therefore, these preliminary results once validated in large patient cohorts could potentially have a significant impact on CRC disease stratification, resulting in a more precise prediction for treatment and treatment outcomes, especially in South African patients.

**Supplementary Information:**

The online version contains supplementary material available at 10.1007/s10528-024-10917-z.

## Introduction

The progression to a global prevalence of inflammatory bowel disease (IBD) spans a 250-year trajectory (Kaplan and Windsor 2021). Initially thought to be confined to high income nations due to ethnic and geographical factors, shifting epidemiological patterns displays an exponential increase in IBD cases particularly in developing nations. Importantly, Sub-Saharan Africa is documented to be the second most populus region in the world potentially leading to a public health challenge in the near future (Watermeyer et al. 2020). IBD can be defined as an idiopathic disorder of the gastrointestinal (GI) tract with two phenotypes; Crohn’s disease (CD) and ulcerative colitis (UC) (Perler et al. 2019). CD leads to inflammation that is known to affect multiple layers of the GI tract (most frequently the ileum). Conversely, UC is characterized by inflammation limited to the mucosal layer of colonic and rectal tissue (Barreiro-de Acosta et al. 2023). Symptoms common to both conditions include diarrhoea, bowel urgency, abdominal pain, GI bleeding and weight loss (Barreiro-de Acosta et al. 2023). The gold standard protocol for the diagnosis of IBD is through an endoscopy (clinical intervention) followed by tissue biopsy and histopathological analysis (Pagnini et al. 2021). These methods are often complemented by radiological imaging approaches including MRI, CT and ultrasonography (Shaban et al. 2022). Following diagnosis and an accurate assessment of disease severity, a treatment decision is made and IBD patients are closely monitored to ascertain the severity of the inflammatory lesions, disease progression and any further complications (Noiseux et al. 2019). Unfortunately, despite the benefits of existing IBD treatments, approximately 10–40% of patients present with primary non-responsiveness (Marsal et al. 2022), with an estimated 23–46% of patients displaying non-responsiveness after a 1-year treatment period (Cai et al. 2021). To combat this resistance, the patient-specific treatment regimens require accurate patient stratification, the use of effective biomarkers and the identification of the best clinical pathways for different individuals (Denson et al. 2019). Additionally, chronic inflammatory exposure of the intestinal mucosa plays a mechanistic role in the development of IBD-related colorectal cancer (CRC) (Stidham and Higgins 2018). Chronic persistent inflammation leads to the production of oxidative stress-induced DNA damage promoting the formation of double-stranded breaks, leading to the accumulation of replicative errors that contribute to the formation of a microsatellite instable phenotype (Muller et al. 2020). The hampered fidelity of DNA integrity further promotes the activation of tumour oncogenes and inactivation of tumour suppressor genes. Driver genes that are frequently activated include *APC, KRAS, P53, PIK3CA, SMAD4, ARID1A* and *MYC* amongst others (Shah and Itzkowitz 2022). Consequently, *P53* loss of heterozygosity is documented in more than 80% of IBD-induced CRC patients and is postulated to be an early event in the transformation to a cancerous state (Muller et al. 2020). Interestingly, loss of function to *APC and KRAS* is only documented in 15% and 20% of cases, respectively, suggesting that IBD-induced CRC does not follow the classical APC inactivation pathway towards carcinogenesis (Muller et al. 2020). Furthermore, the pro-inflammatory environment generated by the host immune response and microbiome further exacerbate transformation to a neoplastic state by inducing epigenetic alterations and consequent silencing of several Wnt/β-catenin-related genes, suggesting their potential role in facilitating CRC development (Shah and Itzkowitz 2022). Due to the strong link between inflammation and CRC development, miR-26b was identified as a possible biomarker for inflammation-associated processes in the gastrointestinal system (Benderska et al. 2015; Farouk et al. 2023). However, the sensitivity and specificity of 12 miRNAs were reported to be 87% and 68.4%, respectively (Coleman and Kuwada 2024), and therefore, further research is required.

Even though IBD-associated CRC accounts for 2% of all CRC cases, it constitutes a major cause of death in IBD patients, amounting to 10–15% of all-cause mortality (Stidham and Higgins 2018). It has been reported that patients with chronic IBD have been found to have a greater chance of developing CRC, where severe complications of long-term IBD are almost universally associated with colitis-associated CRC (Zhou et al. 2023). This is further exacerbated by the severity of inflammation and a family history of CRC (Taylor et al. 2020). It is estimated that for every decade that a patient diagnosed with IBD lives, there is an incremental increased risk of developing CRC, 2.9% after 10 years, 5.6% after 20 years and 8.3% after 30 years (Taylor et al. 2020). From the above, it is evident that the inflammation–dysplasia–carcinoma sequence of IBD-CRC is distinct from the classical–adenoma–adenocarcinoma sequence and confers a poorer prognosis (Porter et al. 2021). This consequently highlights the incipient need to develop accurate means of predicting disease trajectory to mitigate adverse outcomes.

Over the last few decades, various biomarkers for IBD diagnosis have been studied, and some of them are now used in clinical practice. The most commonly employed tests assessing serum biomarkers of IBD include evaluating the levels of C-reactive protein (CRP) and erythrocyte sedimentation rate (ESR) (Alghoul et al. 2022). CRP is an acute phase protein that is often upregulated under pro-inflammatory conditions. Despite its widespread use in IBD diagnosis, CRP is upregulated in the case of autoimmune disorders, infections, cardiovascular diseases, diabetes as well as malignancies (Liu et al. 2022). Similar to CRP, ESR levels are also affected by other inflammatory conditions and may be further influenced by physiological factors such as age, gender and pregnancy status (Alghoul et al. 2022). In terms of faecal-based biomarkers, these constitute faecal leukocyte proteins including calprotectin, calgranulin C, lactoferrin and lipocalin-2, which are commonly employed for IBD diagnosis. Faecal calprotectin and lactoferrin levels are increased due to the increased presence of neutrophils in the GI tract of individuals diagnosed with IBD (Ashton and Beattie 2024). Interestingly, lactoferrin plays a crucial role in regulating immune homeostasis and is upregulated in various inflammatory diseases and cancers (Liu et al. 2022). Additionally, Calgranulin C is crucial to stimulating the NFκβ pathway leading to a pro-inflammatory environment but is also upregulated in arthritis (Nakeeb et al. 2023). Lastly, lipocalin-2 is highly expressed by gut epithelial cells in patients with IBD (Kim et al. 2022), thus challenging the sensitivity and specificity of these markers.

Although existing biomarkers have proven useful in differentiating IBD from functional bowel disease, monitoring disease progression, the prediction of recurrence and therapeutic efficacy, as well as clinical prognosis (Wagatsuma et al. 2021), yet the sensitivity and specificity of these markers are influenced by other co-existing disease modalities, whereas conventional diagnostic methods are often invasive with limited sensitivity and incongruent with disease activity, highlighting the crucial need to identify alternative biomarkers that may be useful for early diagnosis (Dubinsky and Braun 2015; Scarpa et al. 2007). Additionally, there are currently no biomarkers available that can monitor the progression of IBD to CRC. To address this caveat, we focussed on transcription factor (TF)-based markers as they accurately represent the dynamic regulatory mechanisms that control disease-related gene expression and are more sensitive and informative for tracking disease development (Lee et al. 2019), therapy effectiveness and personalized medicine approaches by collecting real-time information about a cell’s reaction to changing conditions, as opposed to static gene markers (Dubitzky et al. 2013). TFs regulate and control various gene sets, hence identifying TFs that specifically regulate genes that are involved in IBD and leading to its progression to CRC could serve as a potential way forward for early diagnosis or prevention. In this study, to predict suitable biomarkers of IBD-related CRC, a curated set of IBD genes was analysed, followed by the prediction of TFs potentially controlling these IBD genes. *In-silico* validation of these biomarkers was subsequently performed from online published databases using IBD and CRC patient samples data. We utilized the combination of IBD and CRC biomarkers to predict the early risk of disease progression from IBD to CRC and performed validation of these biomarkers using in silico methods as well as a small number of human patient-derived specimens.

## Materials and Methods

### Workflow: Identification and in Silico Validation of IBD and CRC Biomarkers

We previously curated IBDDB database (https://www.cbrc.kaust.edu.sa/ibd/) (Khan et al. 2021) of IBD genes. The promoter sets of these genes were obtained and evaluated using Eukaryotic Promoter Database (EPDnew) (http://epd.vital-it.ch) (Dreos et al. 2015). The promoters were subsequently examined for TF binding sites (TFBS), which would serve as a link between genes and the respective TFs that regulates these genes. The promoter sequences’ TFBS were then linked to position frequency matrices (PFMs) in mammalian matrix models using JASPAR database (https://jaspar2020.genereg.net/) (Khan et al. 2018) and OProf tool from EPDnew database. We then predicted CRC progression from IBD by combining the known CRC biomarkers with a selected IBD marker, and validated the selected gene signature biomarker set by employing gene expression atlas database (Papatheodorou et al. 2020) along with preliminary validation in South African tissue samples using RT-qPCR.

### IBD Genes Data Source, Promoter Sets and TFBSs Mapping

We previously manually curated a set of 289 experimentally verified IBD genes, which are published as IBDDB database (https://www.cbrc.kaust.edu.sa/ibd/) (Khan et al. 2021). The IBD genes were analysed to identify (transcription start site (TSS)) in their promoters in EPDnew (https://epd.expasy.org/epd/) for *Homo sapiens*. Promoters were selected using -1000 to + 200 (upstream and downstream from the TSS, respectively) in FASTA format. Subsequently, motif occurrences profile around TSS was generated along with the plot displaying frequency of occurrences of TATA box using Oprof tool under signal search analysis (SSA) server (https://epd.expasy.org/ssa/oprof.php). Position-specific scoring matrices (PSSM) were used to represent the specificities of TF binding to their homologous DNA binding motifs. Position weight matrices (PWMs) or PSSM score of > 85% were selected as a threshold for predicted binding sites for vertebrates in the JASPAR (https://jaspar2020.genereg.net/) database (Khan et al. 2018; Fornes et al. 2020). OPOSSUM version 3.0 was used to predict TFBSs (http://opossum.cisreg.ca/oposusum3/). Selection of potential TFs was based on one-tailed fisher exact probability score and TFBSs profiles with fisher exact test score ≥ 7 was used as a cut-off score (Ho Sui et al. 2007).

### Functional Annotation

Enrichment of IBD genes based on their annotated functions was performed using Database for Annotation, Visualization and Integrated Discovery (DAVID, https://david.ncifcrf.gov) (Dennis et al. 2003) and Kyoto Encyclopaedia of Genes and Genomes (KEGG) database release 100.0, October 1, 2021 (https://www.kegg.jp) (Kanehisa and Goto 2000).

### CRC-Related Biomarkers

Four biomarkers carcinoembryonic antigen (CEA), tissue inhibitor of metalloproteases 1 (TIMP1), cancer antigen 724 (CA724) and cancer antigen 199 (CA199) related to CRC were selected that are either in the clinical practice or have a plethora of published literature emphasizing their role as biomarkers of CRC. These biomarkers were used for in silico validation as explained below and were also subjected to preliminary experimental validation using RT-qPCR on samples collected from South African individuals.

### IN SILICO Validation of Biomarkers, Gene Expression Profiling and Survival Analysis

Datasets Gaedcke colorectal, Grauden’s colon, Hong colorectal and Ki colon (Graudens et al. 2006; Ki et al. 2007; Gaedcke et al. 2010; Hong et al. 2010) from the ONCOMINE database (https://www.oncomine.org/) were used to validate the predicted biomarkers. To assess the difference in gene expression levels between normal and tumour tissues, Colon Adenocarcinoma (COAD) and Rectal Adenocarcinoma (READ) datasets were employed. Additionally, these datasets were also compared to The Cancer Genome Atlas Programme (TCGA) normal tissue database and the Genotype-tissue Expression database (GTEx) (https://www.ebi.ac.uk/gxa/home). The Overall Survival (OS) and Disease-Free Survival (DFS) plots were generated using COAD-READ datasets with survival plot extension tool by GEPIA 2 (http://gepia2.cancer-pku.cn/#analysis). Metatranscriptome Meta-Analysis (IBD TaMMA) platform was used to generate *RUNX1* and *TIMP1* expression graphs in UC, CD and normal samples. The online database included a comprehensive collection of 3,853 publicly available RNA-Seq datasets from 26 independent studies performed on IBD-derived and control samples across different tissues (https://ibd-meta-analysis.herokuapp.com/) (Massimino et al. 2021). The expression levels of *RUNX1* and *TIMP1* in CRC were extracted from 2,137 tumour samples from 17 independent cohorts based on Affymetrix Human Genome U133A Arrays integrated into Kaplan–Meier Plotter (Győrffy 2024).

### Statistical Evaluation of Biomarkers

Various parameters such as sensitivity or specificity of the markers (True Positive, False positive, True Negative and False Negative), Likelihood ratio (LR), Positive Predictive Value (PPV), pre- and post-test odds and receiver operating characteristics (ROC) were calculated (Hoo et al. 2017; Ray et al. 2010; Saah and Hoover 1997; Shreffler and Huecker 2020), and interpretations were made according to Table S1 in Supplementary file 1.

### Combination of Multiple Biomarkers

To calculate the combined sensitivity and specificity of the identified biomarker sets, the following formulae were used (Parikh et al. 2008):

For Sensitivity Test = 

$$1-\left(1-{SEN}_{BIOMARKER 1}\right) X \left(1-{SEN}_{BIOMARKER 2}\right) X \left(1-{SEN}_{BIOMARKER 3}\right)$$

For Specificity Test = 

$$1-\left(1-{SPEC}_{BIOMARKER 1}\right) X \left(1-{SPEC}_{BIOMARKER 2}\right) X (1-{SPEC}_{BIOMARKER 3})$$

Data with CI > 95% were selected for further analysis (Schober et al. 2018).

### Validation of Selected TFs in Patient Colorectal Samples

#### Sample Collection: Normal, IBD and CRC

Patients (IBD and CRC) and for normal samples, the patients undergoing routine colonoscopies and colon-related procedures were consented and small biopsies (2 mm^3^) and cancer tissue were collected at the Wits Donald Gordon Medical Centre (Parktown, Johannesburg) after approval from the human research ethics committee (Medical), University of the Witwatersrand (Clearance Number: M170391, M210233). Signed informed consent forms were obtained from each participant prior to use.

#### RNA Extraction, cDNA synthesis, qPCR

RNA was extracted from patient-derived specimens using the RNeasy Mini Kit (Qiagen, Germany) as per the manufacturers protocol. First strand cDNA synthesis was completed using the RevertAid First Strand cDNA Synthesis Kit (ThermoFisher Scientific, USA) according to the manufacturer’s protocol. RT-qPCR was performed on the CFX96 Touch Real-Time PCR Detection System (Bio-Rad, USA) using the Luna Universal qPCR Master Mix (New England Biolabs, USA) employing appropriate controls, where β-actin (*ACTB*) was used as a house keeping control. Relative changes in mRNA expression were analysed using the 2^−∆∆CT^ method and compared with the healthy control group. Statistical analysis employed included the Kruskal–Wallis test to assess significance across all three patient groups (healthy, IBD and CRC).

## Results

### Enrichment Analysis of IBD Genes

**Fig. 1:**
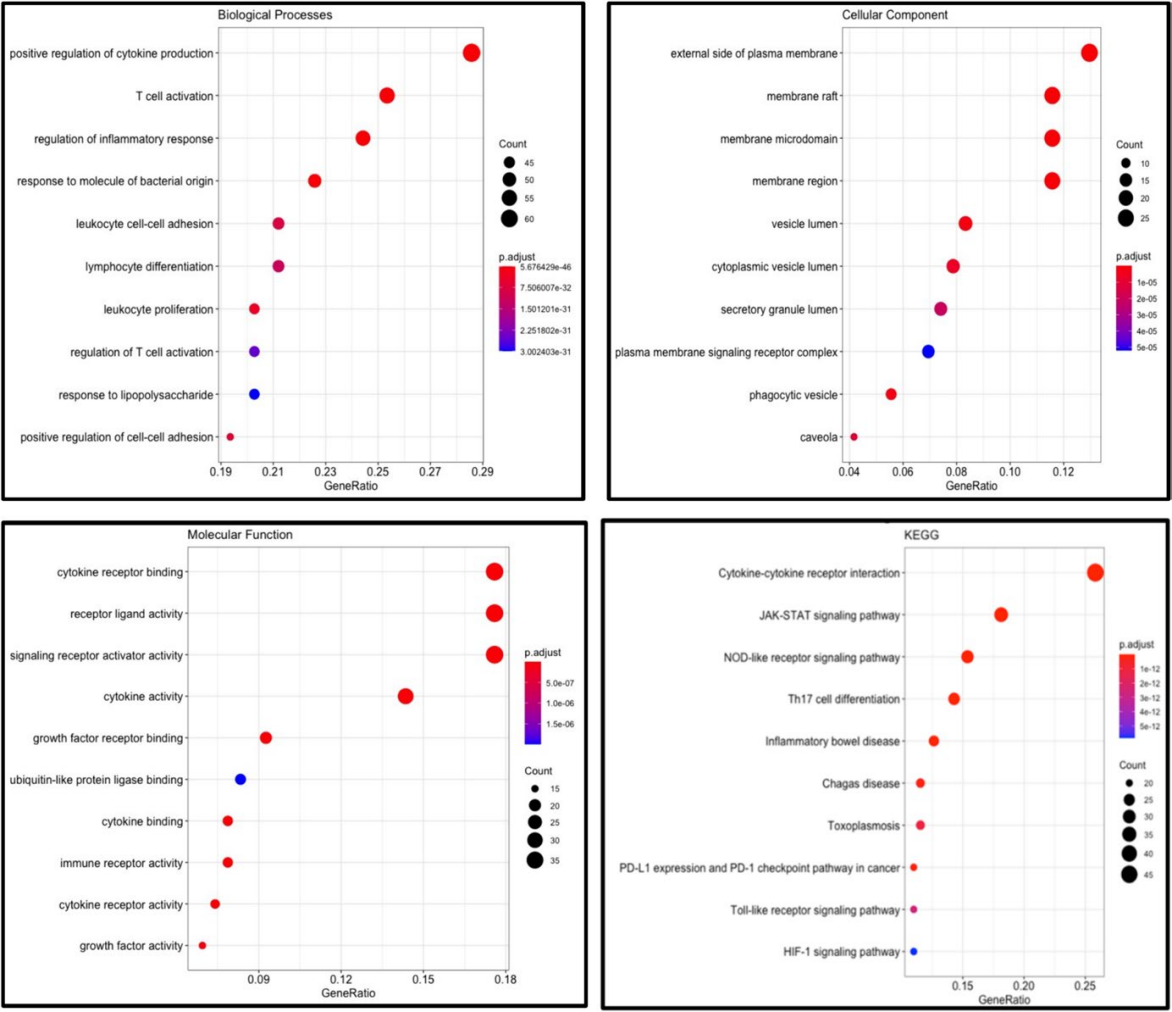
GO annotations and KEGG enrichment Pathway analysis of 289 IBD-related genes Biological processes,Cellular components,Molecular functions and KEGG Pathways found to be enriched. The size of the black circles represents the relative number of genes showing enrichment of a process/pathway. Red/blue bars represent the statistical significance at p < 0.01 for KEGG pathways, whereas p < 005 represents the significance cut-off for GO annotations

Functional enrichment through GO and KEGG pathway analysis was performed on all 289 genes using Cluster profiler (Fig. 1). The enriched pathways with p value < 0.01 are represented in the plots. The most significant terms with p value < 0.05 in biological processes was positive regulation of cytokine production, while inflammatory response, T-Cell activation and adhesion were also enriched. Additionally, cytokine-related molecular processes were found to be enriched, and interestingly, KEGG pathways related to IBD and cancer (Jak-STAT, PD1-checkpoint, and HIF-1 pathways) were enriched. This demonstrates the involvement of multiple pathways intersecting IBD and cancer, and points towards the involvement of IBD-related genes in promoting a cancer phenotype.

### Identification of TFs Regulating IBD Genes as Putative Biomarkers for IBD

A total of 11 TFs were identified to have binding sites (with Fisher score of ≥ 7) in 289 IBD genes (Supplementary file 1, Table S2). Using a combination of a greater number of gene hits and a higher Fisher score, seven biomarkers were selected for downstream testing, and the results are presented in Table 1.

**Table 1:** Top-ranked seven TFs regulating 289 IBD genes sorted by number of gene hits and higher Fisher score (≥ 7) as identified through OPOSSUM3

Transcription factors (TFs)	Gene hits	TFBS hits	Fisher score
SPIB	154	593	11.710
ELF5	136	401	9.706
SPI1	134	341	13.088
FEV	130	309	14.132
RUNX1	106	196	12.942
NFKB1	69	108	11.856
RELA	57	80	13.501

These TFs regulate a large number of IBD-related genes, for example, SPIB regulates 154 out of 289 i.e. 53% of genes, while RUNX1 regulates 106 genes out of 289 genes, i.e. 36% of 289 IBD genes. Thus, the identified TFs can be considered at the hub of the gene network linked to IBD. Therefore, the change in expression level of one TF will affect the network of hundreds of genes linked with a disease.

### Diagnostic Parameters of Predicted IBD Biomarkers

An *in-silico* approach was used to confirm the role of selected TFs in the development of the IBD and its progression through mining of biological databases such as ONCOMINE, TCGA and Gene Expression Atlas. Various parameters (Table 2) were calculated. Out of seven biomarkers, RUNX1 had both high sensitivity and specificity (77% and 66%, respectively) in comparison to other biomarkers (SPIB, ELF5, SPI1, FEV, NFKB1 and RELA). Additional assessments on RUNX1 and other predicted biomarkers of IBD, using various statistical tests such as LR, PPV, NPV, accuracy, pre-test and post-test probability odds, and AUC indicated that RUNX1 scored the highest test values among all the other biomarkers (Table 2) with an AUC_ROC_ value of 0.74, which exceeded the AUC values of the other IBD biomarkers. The PPV value of this biomarker is high, 92% as compared to all the other biomarkers. The LR is used to determine a diagnostic test’s efficacy and to assist in the selection of the optimal diagnostic tests. LR has a significant advantage over sensitivity and specificity in that it is less likely to modify with disorder prevalence and may be used to aggregate the findings of many diagnostic tests to assess the likelihood of a target disorder being diagnosed (Hayden and Brown 1999). The LR for RUNX1 is LR + 2.21 and LR- is 0.38. Here, LR- 0.38 indicates that value 0.38 decreases the probability of disease about 25% but it still presents a disease. As LR- should be between 0 and 0.1 and not 1.0. It also suggests that a person without IBD is about 3 times (= 1/0.38) more likely to have a negative test results than someone with IBD (Mathioudakis et al. 2018). Hence considering all the aspects of a diagnostic biomarker, RUNX1 can be considered as a putative TF-based biomarker of IBD.

**Table 2:** Diagnostic parameters of predicted IBD biomarkers for clinical utility such as Sensitivity, Specificity, PPV, NPV, Pre-test odds, Post-test odds, ${\text{AUC}}_{\text{ROC}}$ and LR at 95% CI

Biomarker	Sensitivity (%)	Specificity (%)	PPV (%)	NPV (%)	Pre-test odds	Post-test odds ( +) [95% CI]:	Post-test odds (−) [95% CI]:	Likelihood ratio ( +) (−)	${\text{AUC}}_{\text{ROC}}$
SPIB	59	3	52	3	0.66	92 [48 – 57%]	2 [[81–100%]	0.60 16.4	0.39 [0.6–1]
ELF5	69	26	63	31	0.45	30 [58 – 69%]	16 [54–81%]	0.93 0.42	0.54 [0.71–0.89]
SPI1	43	55	65	33	1.27	55 [55 – 74%]	56 [58–73%]	0.98 1.02	0.48 [0.71–0.89]
FEV	53	50	80	22	0.88	48 [68 – 88%]	45 [65–87%]	1.06 0.94	0.53 [0.71–0.88]
NFKB1	50	17	70	7	1.00	37 [62 – 78%]	75 [77–98%]	0.6 2.94	0.43 [0.69–0.91]
RUNX1	77	66	92	32	0.33	42 [85 – 97%]	12 [55–79%]	2.21 0.38	0.74 [0.67–0.83]
RELA	81	8	78	8	0.23	17 [85 – 97%]	35 [55–79%]	0.88 2.25	0.66 [0.82–0.98]

### Combination Evaluation of IBD Biomarkers

Although RUNX1 displayed 77% sensitivity and 66% specificity (Table 2), it is still insufficient to stratify patients. Therefore, to improve the accuracy of the diagnosis of IBD, we tested various combinations of the biomarkers as shown in Table 3.

**Table 3:** Evaluation of sensitivity and specificity of various combinations of predicted biomarkers of IBD

Combinations tested	Markers	Sensitivity	Specificity
**Seven Markers**	SPIB + ELF5 + SPI1 + FEV + NFKB1 + RUNX1 + RELA	99.9%	87%
**Five markers, below a and b**			
(a) Excluded: NFKB1, RELA	SPIB + ELF5 + SPI1 + FEV + RUNX1	99%	95%
(b) Excluded: SPI1, FEV	NFKB1 + RUNX1 + RELA + SPIB + ELF5	99%	81%
**Four Markers**	FEV + NFKB1 + RELA + RUNX1	99%	87%
**Three Markers, below a–d**			
(a) Excluded: SPIB, ELF5, SPI1, FEV	NFKB1 + RELA + RUNX1	97%	74%
(b) Excluded: FEV, NFKB1, RUNX1, RELA	SPIB + ELF5 + SPI1	93%	68%
(c) Excluded: RELA, SPIB, ELF5, SPI1	FEV + NFKB1 + RUNX1	95%	86%
(d) Excluded: ELF5, SPI1, NFKB1, RUNX1	RELA + SPIB + FEV	96%	55%

It is clear from Table 3 that the five biomarkers gene signature set (SPIB + ELF5 + SPI1 + FEV + RUNX1) can provide better diagnosis with a sensitivity of 99% and specificity of 95%.

We further validated the expression levels of the predicted seven IBD biomarkers in patient samples through a manual search through Gene Expression Atlas Database (https://www.ebi.ac.uk/gxa/home) as shown in Table 4. This table summarizes the representative expression levels of genes/proteins in various sample types which can be measured using various experimental techniques in both IBD and CRC samples, which can vary among studies included in the database Interestingly, the expression of almost all seven TF biomarkers was found to be increased in IBD while decreased in CRC especially in CRC, depending on the sample type used, e.g. the expression of *RUNX1* was increased in tissues while decreased in platelets. The variable expression in IBD vs. CRC suggests that these biomarkers may have the potential to monitor progression from IBD to CRC. It must be noted here that no single database hosts all the published studies performed on a disease, and the expression values can change depending on the experimental design, sample type, assay technology used, disease state and many other technical procedures involved in the processing and storage of samples. Therefore, the expression values presented here are not claimed to be the absolute true reflection of the expression of predicted biomarkers. However, the other diagnostic statistical parameters shown above (Table 2) demonstrate that *RUNX1* has the best diagnostic capabilities. Therefore, *RUNX1* was combined with four well-known CRC biomarkers to test if a gene signature biomarker set can be developed for further testing to predict the progression from IBD to CRC.

**Table 4:** Expression of IBD biomarkers in patients’ samples extracted from the Gene Expression Atlas Database, where WBC = White blood cells, IHC = Immunohistochemistry, CD = Crohn’s Disease and UC = Ulcerative Colitis

Biomarker	Expression Log_2_-Fold Change (IBD)	IBD type	IBD Sample type	Expression Log_2_-Fold change (CRC)	CRC sample type	Other Routinely used detection techniques from literature
SPIB	2.7 [Up]	CD	Bone marrow/ WBC	−2.2 [Down]	Tissue	qPCR/IHC/Western blotting
ELF5	1.5 [Up]	CD	Intestinal biopsies/WBC	−3.2 [Down]	Tissue	qPCR/IHC/Western blotting
SPI1	1.8 [Up]	UC, CD	Blood /intestinal biopsies	−2.0 [Down]	Blood	qPCR/IHC/Western blotting
FEV	1.2 [Up]	UC	Tissue /Blood	−1.4 [Down]	Tissue	qPCR/IHC/Western blotting
NFKB1	1.7 [Up]	UC	WBC/Macrophages	−1.1 [Down]	Serum/ Serrated polyposis	qPCR/IHC/Western blotting
RUNX1	1.3 [Up]	CD	Tissue/Blood	1.5 in tissue [up] to -1.6 in platelets [Down]	Tissue/platelets	qPCR/IHC/Western blotting
RELA	1.4 [Up]	CD	Blood and intestinal biopsies	−1.9 [Down]	Tissue	qPCR/IHC/Western blotting

### Potential Biomarkers to Predict Progression from IBD to CRC

Four known CRC biomarkers (CEA, TIMP1, CA724 and CA199) were subjected to diagnostic parameter analysis and the results are shown in Table 5. CEA had a diagnostic sensitivity of 46% and specificity of 80%. TIMP1 demonstrated a sensitivity of 52% and specificity of 60%, while CA724 and CA199 had sensitivity of 45% and 14.39%, and specificity of 97% and 89%, respectively (Table 5). Overall, their accuracy was comparatively lower than RUNX1, but had reasonably higher PPV and LR for some of these markers. Therefore, we looked at the expression patterns of these four biomarkers along with RUNX1 in the IBD and CRC patient samples as shown in Table 6. There was a distinct difference in serum levels as well as gene expression levels in IBD vs CRC samples. Since the individual diagnostic capability of each marker is limited, this suggests that the combination of these biomarkers may have the potential to enhance diagnostic accuracy and provide information about the progression of IBD from CRC.

**Table 5:** Diagnostic parameters of predicted IBD to CRC biomarkers for clinical utility such as Sensitivity, Specificity, PPV, NPV, Pre-test odds, Post-test odds, ${\text{AUC}}_{\text{ROC}}$ and LR at 95% CI

Biomarker	Sensitivity (%)	Specificity (%)	PPV (%)	NPV (%)	Pre-test odds	Post-test odds ( +) [95% CI]:	Post-test odds (−) [95% CI]:	Likelihood Ratio ( +) (−)	${\text{AUC}}_{\text{ROC}}$
RUNX1	75	66	92	32	0.33	42% [85–97%]	12% [55–79%]	2.2 0.3	0.74 [0.67–0.83]
CEA	46	80	89.7	28.4	1.15	73% [84–93%]	43.2% [68–75%]	2.33 0.67	0.53 [0.44–0.65]
TIMP1	52	60	42.2	69.5	0.92	54% [34–51%]	42.5% [24–38%]	1.3 0.8	0.57 [0.50–0.71]
CA724	45	97	98.4	31.9	1.22	95% [94–100%]	41% [66–70%]	16.6 0.57	0.56 [0.50–0.70]
CA199	14.4	89	83.7	21.7	5.94	88.6% [72–91%]	85.1% [77–80%]	1.36 0.59	0.30 [0.17–0.43]

**Table 6:** Expression profiles of predicted IBD to CRC biomarkers in patients’ samples obtained from the published literature and the Gene Expression Atlas Database. Where FPD = Familial Platelet Disorder, IHC = Immunohistochemistry, iFOBT = immunochemical Faecal occult blood test

Biomarker	Expression Log_2_-Fold change (IBD)	Serum level in IBD	Expression Log _2_-Fold change (CRC)	Serum level in CRC	Samples	Other Routinely used detection techniques from literature
RUNX1	1.3 [Up]	Blood Platelets (proposed link between Reactive Thrombocytosis in FPD& Inflammation) > 450 X 10^5^ μL (Danese et al. 2004; Hayashi et al. 2017) but no measured values of RUNX1 found	1.5 in tissues [up] to -1.6 in platelets [Down]	Abnormality in blood platelets but no measured values found (Voora et al. 2016)	Tissue/blood platelets	PCR/RT-qPCR/IHC/Western Blot
CEA	4.2 [Up]	< 2.5 ng/mL (Wannhoff et al. 2017)	4.0 [Up]	> 5 ng/mL (Su et al. 2012)	Blood serum	IHC/ iFOBT
TIMP1	4.3 [Up]	< 450.5 ng/mL (Kapsoritakis et al. 2008)	2.6 [Up]	85.9–100.8 ng/mL (Christensen et al. 2015)	Blood serum	IHC/ iFOBT
CA724	2.0 [Up]	0–6 U/mL (Liang and Yang 2022)	3.5 [Up]	0.8 IU/mL (Cao et al. 2023)	Blood serum	IHC/ iFOBT
CA199	−1.2 [Down]	> 37 U/mL (Tobi et al. 1991)	1.3 [Up]	> 500 IU/mL (Tobi et al. 1991)	Blood serum	IHC/ iFOBT

### Combination of a Predicted IBD Biomarker (RUNX1) with CRC Biomarkers to Predict Progression to CRC

Various combination sets and their sensitivity and specificity values are shown in Table 7, from which it can be inferred that the combination of a four-marker set (CEA + TIMP1 + CA724 + RUNX1) displayed similar diagnostic capability relative to the five-markers set (CEA + TIMP1 + CA724 + RUNX1 + CA199). Therefore, to reduce the cost and time of testing, the four-marker set may be employed without compromising the diagnostic capabilities. To establish this further, survival analysis was performed.

**Table 7:** Evaluation of sensitivity and specificity of various combinations of RUNX1 (a predicted biomarker of IBD) with known CRC markers

Combinations	Markers	Sensitivity	Specificity
Five Markers	CEA + TIMP1 + CA724 + RUNX1 + CA199	97%	99%
Four Markers (a) (Excluded: CA199) (b) (Excluded: TIMP1)	CEA + TIMP1 + CA724 + RUNX1	96.5%	99.9%
	CEA + CA724 + RUNX1 + CA199	94%	99%
Three Markers (Excluded: TIMP1, CA199)	CEA + CA724 + RUNX1	93%	99.7%

**Fig. 2:**
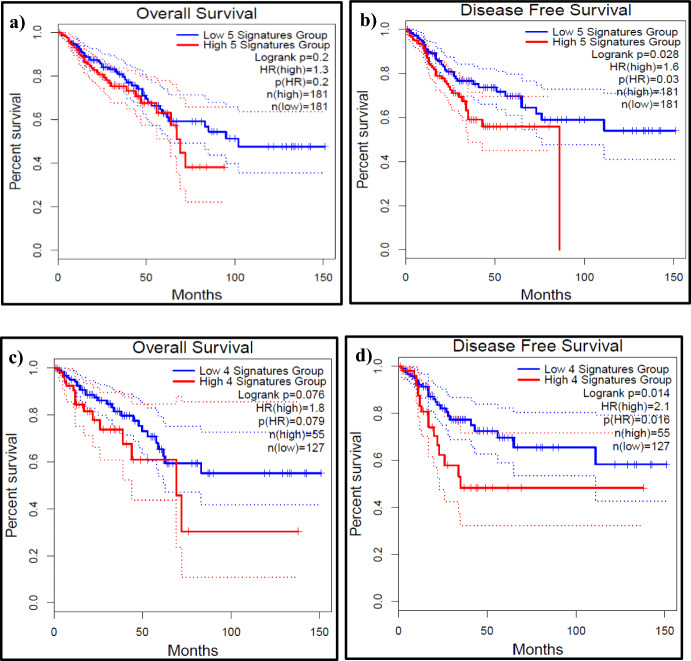
Kaplan–Meier Plots depicting OS and DSF for the 5 biomarkers gene signature set and 4 biomarkers gene signature set The combination of 5 biomarkers (CEA + TIMP1 + CA724 + RUNX1 + CA199) expression was associated with **a** worse OS, hazard ratio (HR) = 1.3, log rank P = 0.2 and **b** worse DFS, HR = 1.6, log rank P = 0.028. The combination of 4 biomarkers gene set (CEA + TIMP1 + CA724 + RUNX1) expression was associated with **c** worse OS, hazard ratio (HR) = 1.8, log rank P = 0.2 and **d** worse DFS, HR = 2.1, log rank P = 0.014

To test the effectiveness of 4 biomarkers gene signature vs. 5 biomarkers gene signature on OS and DFS of the patients, GEPIA2 was used to generate Kaplan–Meier plot**s** using CRC COAD-READ datasets. The 5 biomarkers grouped together as a signature (multi-gene) showed that patients with high expression of these biomarkers were primarily associated with shorter OS (*p* value = 0.2) and short DFS (*p* value = 0.028) as shown in (Fig. 2 a, b). In comparison, 4 biomarkers gene signature set (CEA + TIMP1 + CA724 + RUNX1) was also associated with shorter OS (*p* value = 0.076) with short DFS (*p* value = 0.014) (Fig. 2 c, d). These data suggest that 4 markers gene set may act as a predictor of CRC and survival in patients.

### Preliminary Experimental Validation in South African Patient Samples

**Fig. 3:**
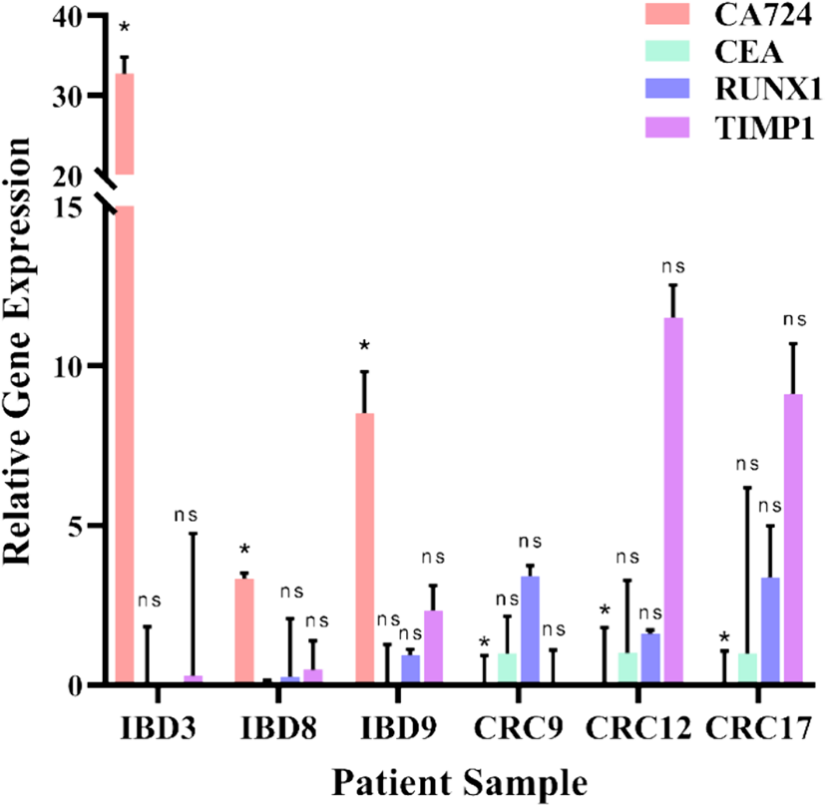
In vitro validation of in silico markers identified using South African Patient samples Relative gene expression profile of *CA724, CEA, RUNX1* and *TIMP1* in IBD (*n* = 3) and CRC patients (*n* = 3) obtained by RT-qPCR analysis. Endogenous expression of these genes was employed to normalise the data to the healthy patient group (*n* = 3). Data represent the mean ± standard deviation (S.D) (*n* = 6). The Kruskal–Wallis test was employed for statistical analysis to assess significance across all three patient groups (healthy, IBD and CRC) which proved a significant difference does exist between these groups, where *p* < 0.05 (*) and ns is non-significant as represented above

Following the in silico identification of the 4 biomarkers gene signature set (CEA, TIMP1, CA724 and RUNX1), RT-qPCR was performed to validate these findings (Fig. 3). RNA was extracted from patient tissue specimens obtained from IBD-diagnosed subjects (*n* = 3) and CRC diagnosed subjects (*n* = 3), which were subsequently normalised to the healthy group (*n* = 3). The Kruskal–Wallis test was employed for statistical analysis to assess significance across all three patient groups (healthy, IBD and CRC) which proved a significant difference does exist between these groups. Importantly, relative gene expression analysis indicated that *CA724* was exclusively expressed at a significantly higher levels in the IBD patient group when compared to the CRC group (*p* < 0.05), thus suggesting that *CA724* can act as diagnostic biomarker for IBD. In contrast to this, *CEA* was sufficiently expressed in the CRC patient group with very low levels detectable in the IBD patient group. Interestingly, both *RUNX1* and *TIMP1* were commonly expressed in both groups with higher levels detected in the CRC group. These results show that when combined, these markers can help to predict progression of IBD to CRC through regular monitoring (once a year), where reducing expression of *CA724* in an IBD patient combined with increasing expression of *RUNX1* and *TIMP1* may be an alarm to perform additional testing for CRC, whereas expression of CEA may help confirm onset of an early CRC phenotype.

### Validation of *RUNX1* and *TIMP1* Expression in Large Patient Cohorts Through In Silico Analysis

**Fig. 4:**
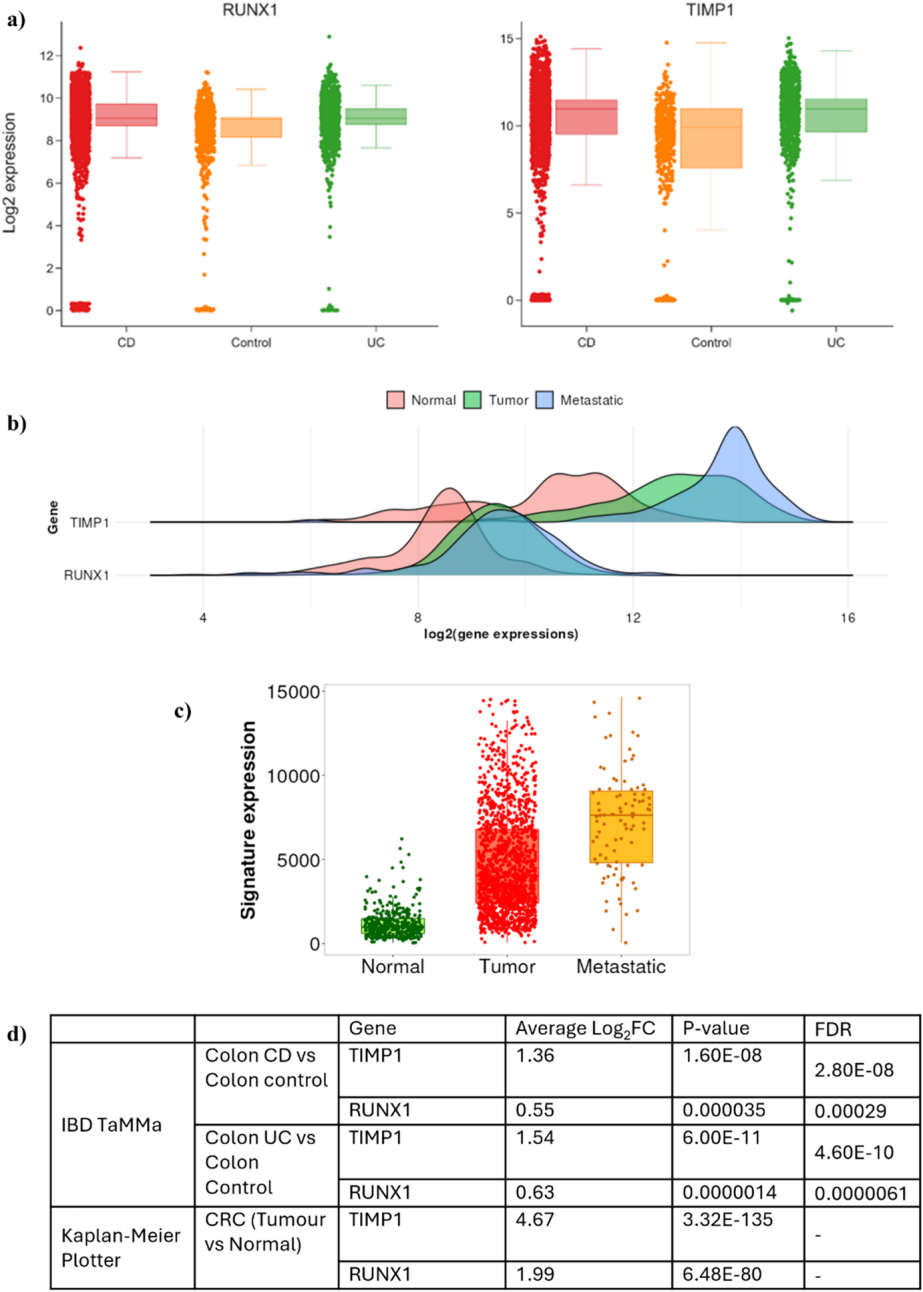
Validation of biomarkers in IBD (UC and CD) samples using IBD TaMMa database and in CRC samples using Kaplan–Meier Plotter. Human IBD data was queried in the IBD TaMMa database for disease groups (CD and UC) vs. control normal samples, and **a** Log_2_ expression values were plotted for genes *RUNX1* and *TIMP1* using FDR<1^-10^ as a default cut-off. **b** represents the expression profiles of *RUNX1 *and *TIMP1* in normal and CRC (tumour and metastatic) samples, whereas **c** showcases the signature expression analysis (*RUNX1+TIMP1*) which calculates the means of the selected gene signature across each patient sample one by one in the database, **d** shows the comparison of Log_2_FC in IBD and CRC samples using two databases. *FDR*, false discovery rate, *CD* Crohn’s disease, *UC* ulcerative colitis, *IBD* inflammatory bowel disease, *CRC* colorectal cancer, *FC* fold change

Due to the small patient sample size (Fig. 3) used for the testing of predicted biomarkers, we further performed additional in silico analysis using previously published IBD and CRC datasets housed in various online databases. For analysis of biomarkers in IBD, we used Metatranscriptome Meta-Analysis (IBD TaMMA) platform (Massimino et al. 2021). Two of the biomarkers *CEA* and *CA724* were not present in this database, whereas *TIMP1* was found to have slightly higher expression than *RUNX1* in the colon of UC and CD samples, and their overall expression was also slightly higher than the control samples (Fig. 4a).

To evaluate expression levels in CRC, the combined colon cancer database integrated into Kaplan–Meier Plotter (Győrffy 2024) was mined, which includes data from 2,137 tumour samples from 17 independent cohorts, based on Affymetrix Human Genome U133A Arrays. Figure 4b shows the density plot of expression patterns of *TIMP1* and *RUNX1* genes in normal, tumour and metastatic samples. The plot demonstrates an increase in expression levels of the genes as the disease progresses. Figure 4c shows the expression levels of both genes combined (signature expression *RUNX1* + *TIMP1*), where tumour samples have 4.17 Log_2_FC in expression vs normal, and metastatic samples have 1.87 FC in expression vs tumour samples with Kruskal Wallis *p* value = 1.08^–148^. It is clear from the data presented in Fig. 4d, that expression of *RUNX1* and *TIMP1* is slightly higher in IBD patients as compared to normal, whereas the expression of both genes increases three- to fourfolds as CRC develops and progresses and follow the following expression pattern: normal < IBD < tumour < metastasis, thus providing a capability to monitor progression of IBD to CRC.

## Discussion

In this study, a bioinformatics approach was employed to identify putative TF biomarkers for the early diagnosis of IBD as well as biomarkers that can predict progression from IBD to CRC. We identified seven TFs i.e. SPIB, FEV, ELF5, SPI1, NFKB1, RUNX1 and RELA through our in silico investigations, and validated these in publicly available gene expression patient datasets by assessing their performance through various diagnostic parameters. We identified RUNX1 as a promising TF-based biomarker for IBD because of its high sensitivity and specificity as 77% and 66%, respectively, in comparison to other biomarkers. Additionally, *RUNX1* had TF binding sites in 106 out of 289 curated IBD genes, thus indicating its ability to regulate expression of 36% of all investigated IBD genes. RUNX1 is a tumour suppressor and is crucial for the formation of the GI tract’s epithelium and for maintaining the balance of the intestinal stem/progenitor cell population (Chuang et al. 2020). Importantly, the combination of *RUNX1* with four additional biomarkers, namely, *SPIB, ELF5, SPI1* and *FEV*, enhanced diagnostic accuracy exhibiting a sensitivity of 99% and specificity of 95% (Table 3). SPIB also displays tumour suppressive activity which is facilitated by the activation of NFκβ and JNK signalling (Zhao et al. 2021). On this basis, the upregulation of SPIB is justified based on the crucial role of the NFκβ and JNK pathways in the induction of the expression of pro-inflammatory cytokines and chemokines stimulating an inflammatory response (Liu et al. 2017; Zhao et al. 2016). The identification of ELF5 as a potential biomarker is quite a novel finding and may be justified by a recent study documenting demethylation in the promoter regions of ELF5 when modelling chronic submergence damage that leads to an IBD state (Rees et al. 2022). It should be noted that ELF5 belongs to ELF family of ETS family of TFs whose binding status is inherently sensitive to the methylation status of the sequence (Yin et al. 2017). Moreover, SPI1 has known roles in the regulation of cytokine signalling and was also seen to be significantly upregulated in a European cohort of individuals diagnosed with IBD when compared relative to the controls (Nowak et al. 2022). A novel finding of a recent study (Nowak et al. 2022) implicates SPI1 as a key regulator of Th9 immunity, where Th9 cells are known to induce several inflammatory diseases including IBD through the production and secretion of IL-9 (Vyas and Goswami 2018). Interestingly, the emergence of FEV as a potential IBD biomarker requires further investigation as the conventional role of this TF is in the serotonergic pathway and neurodevelopment (Kriegebaum et al. 2010). Moreover, deregulated expression, transcriptional activity or chromosomal translocation implicate FEV in carcinogenesis (Cooper et al. 2015), which might make it an interesting marker to further incorporate in a biomarker panel studying the progression of IBD to CRC.

IBD patients with long-term severe inflammation of the gut are more likely to acquire CRC (Majumder et al. 2022). UC patients had a risk of 5.7 times that of the general population, according to population-based research (Mann et al. 2022). To identify a set of biomarkers suitable for monitoring progression of IBD to CRC, RUNX1 was further tested in combination with four known CRC biomarkers (CEA, TIMP1, CA724, CA199). These markers were tested in various combinations (Table 7), where the set of 4 biomarkers (CEA + TIMP1 + CA724 + RUNX1) displayed an increased sensitivity of 96.5% and increased specificity of 99.9%. This set of 4 biomarkers displayed a similar diagnostic capability to the set of 5 biomarkers and was consequently validated by employing patient-derived specimens from South African individuals diagnosed with IBD or CRC.

CEA is an oncofetal antigen that indicates the existence of a variety of tumours (Kankanala and Mukkamalla 2022). It is often detected in blood at very low levels in non-diseased adults (Hao et al. 2019). Increased CEA levels are linked to cancer, which makes it a potential biomarker for prognostic and diagnostic evaluations in CRC patients (Ma et al. 2022; Ming-Sheng et al. 2022). In our study, *CEA* was seen to be almost exclusively expressed in CRC patients indicating that it could serve as a good diagnostic marker for CRC patients. This corresponds well to previous studies where preoperative CEA levels can act as an independent prognostic marker for stages I-III in CRC, and can further predict the early risk of CRC in UC patients (Zhen et al. 2018). Furthermore, preoperative CEA assessment is beneficial since it can provide autonomous prognostic information, assist with surgical administration and serve as a baseline for later findings (Xie et al. 2022). It is recommended that CEA levels should be assessed every few months at least for five years after diagnosis in patients with CRC stage II or III illness who may be candidates for liver resection (Nicholson et al. 2015). Matrix metalloproteinases (MMPs) and TIMP1 are the main enzymes responsible for regulating collagen degradation and extracellular matrix remodelling (Qin et al. 2021). TIMP1 can regulate apoptosis, angiogenesis and proliferation in an MMP-independent manner and plays a role in CRC carcinogenesis, according to multiple compelling findings (Tuomisto et al. 2019). Many studies show that TIMP1 can be used as a prognostic as well as a diagnostic biomarker for CRC patients (Macedo et al. 2022; Meng et al. 2018). This corresponds well to data obtained in the current study where *TIMP1* expression is drastically increased in CRC patients. Furthermore, the in silico analysis (Fig. 4) follows the same trend of expression profiles of *TIMP1* as shown in the patient samples, thus further validating the diagnostic capability of the investigated biomarkers in this study. TIMP1 sustains the major diagnostic and prognostic implications for the progression of CRC, particularly, serving as a useful marker for differentiating CRC from colorectal adenomas (Łukaszewicz-Zając and Mroczko 2021). It was reported that the intestinal mucosa of patients with UC exhibited notably diminished levels of TIMP1 which is well represented in our study. Interestingly, a strong correlation between TIMP1 expression and a number of immune cell markers was established (Pan et al. 2023) which could potentiate employing this marker as a possible indicator of immunotherapy responsiveness.

CA199 and CA724 are found on the surface of many cancer cells, including those with hepatocellular carcinoma, ovarian cancer, gastric cancer and CRC. CA199 antigen is identified as a tumour marker recognized as a diagnostic, prognostic and as a surveillance marker for CRC (Yamashita and Watanabe 2009; Kuang et al. 2020). The monoclonal antibody NS19-9, is used in clinical settings as a traditional tumour marker against CA-199 (Thurin 2021). Despite increased levels of CA724 in several cancers, many emerging studies are now documenting conflicting results as to the reliability of employing this marker for CRC diagnosis as this marker is not a unique product of cancer cells. In particular, CA724 is significantly increased in inflammatory diseases such as gout (Zhang et al. 2019), gastric ulcers and polyps, and also atrophic gastritis (Hu et al. 2019) and may consequently justify the increased expression of CA724 in IBD patients. This marker is seen to be influenced by regional variation (Jing et al. 2019) and may potentially explain the mismatch in the in silico data and in vitro data obtained. This finding, however, is preliminary and should be validated on a larger patient cohort. Consequently, the ability of CA724 to classify IBD patient may potentially be restricted to South African populations. Expression of RUNX1 seems to vary from study to study where it has been found to be downregulated (Wu et al. 2020), or upregulated in CRC tissues (similar to our preliminary validation as well as the in silico validation (Fig. 3, 4) and its variants to be associated with increased risk for colon and rectal carcinogenesis (Zhou et al. 2017).

Consequently, factoring in the limitations of each of these biomarkers individually, it can be stated that none of these markers may be used in isolation to accurately predict IBD to CRC progression and our study posits that the combination of these biomarkers has the potential to greatly improve the diagnostic accuracy. Additionally, these biomarkers could also be used to monitor the progress of CRC from tumour development to metastasis. It will be intriguing to investigate the effect of chemotherapeutic drugs on the expression profiles of these two markers in the IBD and CRC patients.

IBD and its progression to CRC need to be further explored due to its multifaceted nature. This study further highlights the translational implications of European population data to African populations and points out the incipient need to explore African disease-specific biology using a larger cohort of patients in an IBD—CRC context in order to identify reliable and accurate diagnostic markers. Findings obtained further reinforce the fact that the use of individual markers to predict IBD to CRC diagnosis is unreliable due to their low sensitivity (Zhang et al. 2023) and a combination of markers instead proves more effective in enhancing accuracy. Based on the nature of molecular genetics research, both sporadic and hereditary CRC are caused by certain unfavourable mutations. Consequently, it is suggested that screening tests should be more sensitive and precise than currently unavailable screening tests, and confirmation of a putative biomarker set will offer genetic information on malignancy and metastatic progression (Singh et al. 2017).

### Limitations and Strengths

Study strengths include that this is the first study to explore TF-based biomarkers in a South African patient group to infer IBD to CRC progression. The study emphasizes the potential clinical utility of tested biomarkers to have a significant impact on patients’ health care specifically in regions with less available resources. While computational approaches and in silico studies along with preliminary validation in patient samples are useful for preliminary screening, additional validation is required by employing a greater sample size to verify the obtained findings and the clinical application of these biomarkers to the broader South African population and globally. Although the study finds possible diagnostic biomarkers, it does not investigate their function in therapeutic monitoring or treatment decisions, which is a crucial area for future research. In addition, despite the *in-silico* validation demonstrated a clear shift in the expression of *RUNX1* and *TIMP1* between UC and CD in comparison to the normal tissue, due to our limited sample size and medical information, it hindered the comparison of data between UC and CD within our IBD samples and thus could be an interesting avenue to explore in the future. Furthermore, increasing research has shown that IBD is being detected more in younger populations, and therefore, it would be worthwhile validating our findings in a younger patient cohort. In terms of the ability to predict TFBS, this depends on the TF models that are available. We hope that as additional verified models are added to databases like JASPAR, the prediction will become more accurate in the future. Nonetheless, the study highlights the importance of combining several biomarkers to develop panels of gene signature that would provide higher sensitivity and specificity of testing as compared to individual markers.

## Conclusion

In summary, this study has identified promising TF-based biomarkers for the early detection of IBD and for predicting the progression from IBD to CRC. Notably, when assessed by in silico analysis and then validated in patients’ samples, *CEA* + *TIMP1* + *CA724* + *RUNX1* demonstrated excellent diagnostic potential. Further validation of these biomarkers was performed in a small South African cohort. Despite this observation, the expression trends obtained through the patient samples could potentially serve as a non-invasive means of predicting disease progression when performed using blood and could serve as a predictive biomarker panel following validation in a larger patient cohort. Consequently, when fully validated in the future, the knowledge provided by this study can offer clinicians and patients more convenience to diagnose IBD and CRC in both adults and children without the need to undergo invasive endoscopic procedure and bowel preparation prior to the surgery. From an economic standpoint, this biomarker panel can serve as a cost-efficient tool improving access to the developing countries.

## Supplementary Information

Below is the link to the electronic supplementary material.

Supplementary file1 (DOCX 19 KB)

## Data Availability

All data is available from the authors upon request.
