# Supplementary material for: Identification and Validation of Biomarkers to Predict Early Diagnosis of Inflammatory Bowel Disease and Its Progression to Colorectal Cancer
Source: Biochem Genet. 2024 Sep 26;63(4):3717–43. doi: 10.1007/s10528-024-10917-z (PMC12271286; doi:10.1007/s10528-024-10917-z)
Supplement: Supplementary file 1 — Supplementary file1 (DOCX 19 KB) [file 10528_2024_10917_MOESM1_ESM.docx]

Editor correction not required

Since editor corrections are not mandatory for this journal we are forwarding this article for online publication.

Regards
Springer Corrections Team
Scientific Publishing Services
No.6 & 7, 5th Street, Radhakrishnan Salai Mylapore, Chennai, Tamilnadu.
India, Pincode 600 004
